# Inhibition of DNA Repair Enzymes as a Valuable Pharmaceutical Approach

**DOI:** 10.3390/ijms24097954

**Published:** 2023-04-27

**Authors:** Konstantin P. Volcho, Olga I. Lavrik

**Affiliations:** 1 N. N. Vorozhtsov Novosibirsk Institute of Organic Chemistry, Siberian Branch of the Russian Academy of Sciences, 9, Akademika Lavrentieva Ave., 630090 Novosibirsk, Russia; 2 Institute of Chemical Biology and Fundamental Medicine, Siberian Branch of the Russian Academy of Sciences, 630090 Novosibirsk, Russia

The DNA repair system plays a crucial role in maintaining the integrity of the genome. Disturbances in the function of certain DNA repair system enzymes can lead to various diseases and contribute to carcinogenesis. On the other hand, tumor cells are often characterized by the hyperactivity of DNA repair system enzymes, which allows them to resist chemotherapy and radiotherapy for cancer aimed at damaging the DNA of these cells. The ability of cancer cells to recognize DNA damage and initiate DNA repair is one of the key mechanisms for therapeutic resistance of tumors to chemotherapy. Therefore, the targeting of DNA repair enzymes can be used as a strategy to potentiate the cytotoxicity of the currently available DNA damaging agents toward cancer cells. The role of inhibition of DNA repair enzymes in cancer therapy is thoroughly analyzed in the review written by Lodovichi et al [1].

The understanding of the ways DNA repair enzymes function in the repair processes is the basis for creating any therapeutic agents aimed at inhibiting these enzymes and protein factors. One such protein factor is the fused in sarcoma (FUS), an RNA binding protein which has not yet been used as a pharmacological target. FUS interacts with poly(ADP-ribose) produced by PARP1/2 on DNA damages. This process is pivotal for the formation of nonmembrane compartments to process DNA repair. Sukhanova et al. reviewed the participation of FUS in major DNA repair pathways [2], with an emphasis on the interaction of FUS with poly(ADP-ribose) polymerase 1 (PARP1) during poly(ADP-ribosyl)ation events important for the compartmentalization of DNA strand breaks and DNA repair proteins. Molecular mechanisms of action of human apurinic/apyrimidinic endonuclease 1 (APE1), an important enzyme involved in base excision DNA repair, was studied by Moor et al. [3].

The Special issue highlights the pharmacological effects of PARP inhibitors. PARPs are important for repairing single-strand breaks in DNA. Tumors mutated through alternative pathways of repair (BRCA1, BRCA2 or PALB2) are especially sensitive to PARP inhibitors. It is worth noting that some inhibitors of PARP1, including Olaparib, Rucaparib, Niraparib and Talazoparib, are already clinically utilized as antitumor drugs. The review by Maluchenko et al. considers the mechanisms of PARP1 participation in the development of various pathologies, and summarizes data on the effect of natural polyphenols on PARP-dependent cellular processes [4]. An article written by Nilov et al. [5] describes studies in the mechanism of action of 7-methylguanine, a natural inhibitor of PARP1. Sherstyuk et al. demonstrated that new morpholino-nucleoside adenosine dinucleotides form a novel class of pan-PARP inhibitors, which are active against PARP1, PARP2 and PARP3 [6]. In the research by Ryu et al. [7], PARP1 and PARP2 inhibitor *N*-(3-(hydroxycarbamoyl)phenyl)carboxamide (KJ-28d) have been indicated to act as sensitizers of non-small cell lung cancer cell lines to radio- and chemotherapy. At the same time, the PARP1 inhibitor, Olaparib, did not potentiate a cytotoxic effect of chemotherapeutic agent bleomycin in VERO cells (Perini et al. [8]).

Tyrosyl-DNA phosphodiesterase 1 (TDP1) plays a key role in the removal of DNA damage resulting from the poisoning of topoisomerase 1, with clinically important anticancer drugs irinotecan and topotecan. Novel structural type of TDP1 inhibitors was developed based on natural alkaloid berberine by Gladkova et al. [9]. It was shown that these inhibitors are able to increase the cytotoxicity of topotecan against the HeLa cancer cell line. Another type of TDP1 inhibitors was designed based on 4-arylcoumarin conjugated with monoterpene moieties (Khomenko et al. [10]). The new inhibitors induced a significant increase in the antitumor effect of topotecan on the Krebs-2 ascites tumor model in mice. Dyrkheeva et al. showed that thioethers of natural usnic acid demonstrated good inhibitory activity against three DNA repair enzymes, TDP1, TDP2 and PARP1 [11]. Moreover, the synergy of the leader compound with topotecan on HeLa cells was shown.

The importance of nucleotide excision repair (NER) in the formation of tumor cell resistance to cisplatin is reviewed by Duan et al. [12]. Furthermore, Fidrus et al. demonstrated that inhibitors of NER can decrease UVB-irradiation-induced mutagenesis [13].

AKT is involved in the regulation of DNA damage response and repair. Boichuk et al. found that the inhibition of the AKT signaling pathway sensitizes tumors to anticancer drug, Doxorubicin, by targeting homology-mediated DNA reparation [14].

Finally, in their review, Mechetin et al. demonstrated that the inhibition of DNA glycosylases, the enzymes that initiate the base excision repair (BER) pathway, could be useful not only in the treatment of cancer, but also to cure neurodegenerative diseases, chronic inflammation, as well as bacterial and viral infections [15].

Thus, the articles and reviews published in this Special Issue emphasize the importance of DNA repair enzyme inhibition in the fight against various diseases, with major attention paid to the development of anticancer drugs, as well as the fast progress in this field.
